# Supplementary figures and images for: The effect of soil sample size, for practical DNA extraction, on soil microbial diversity in different taxonomic ranks
Source: PLoS One. 2021 Nov 18;16(11):e0260121. doi: 10.1371/journal.pone.0260121 (PMC8601499; doi:10.1371/journal.pone.0260121)

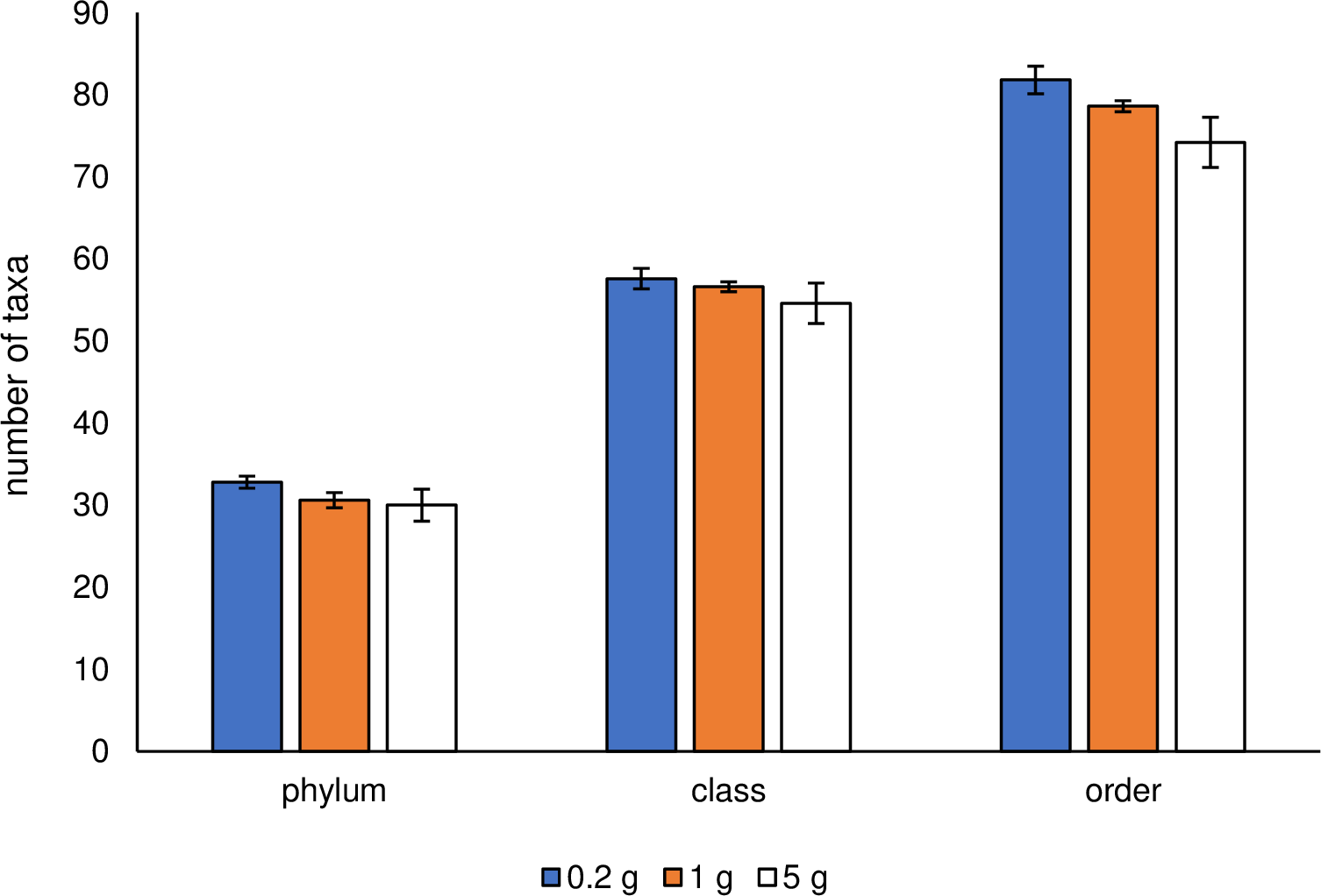

Supplement: S1 Fig — Error bars represent standard errors. (TIF) [file pone.0260121.s001.tif]

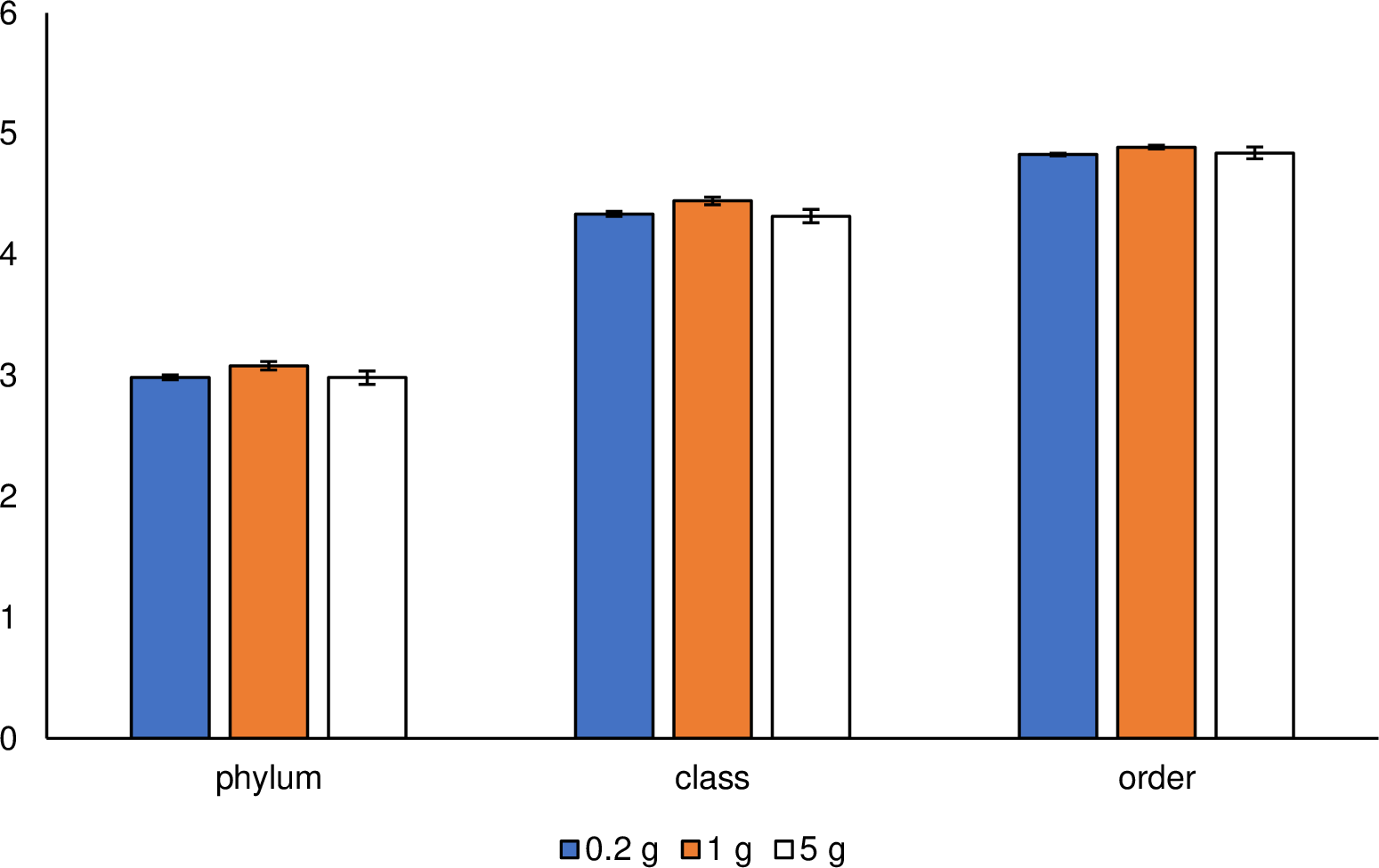

Supplement: S2 Fig — Error bars represent standard errors. (TIF) [file pone.0260121.s002.tif]

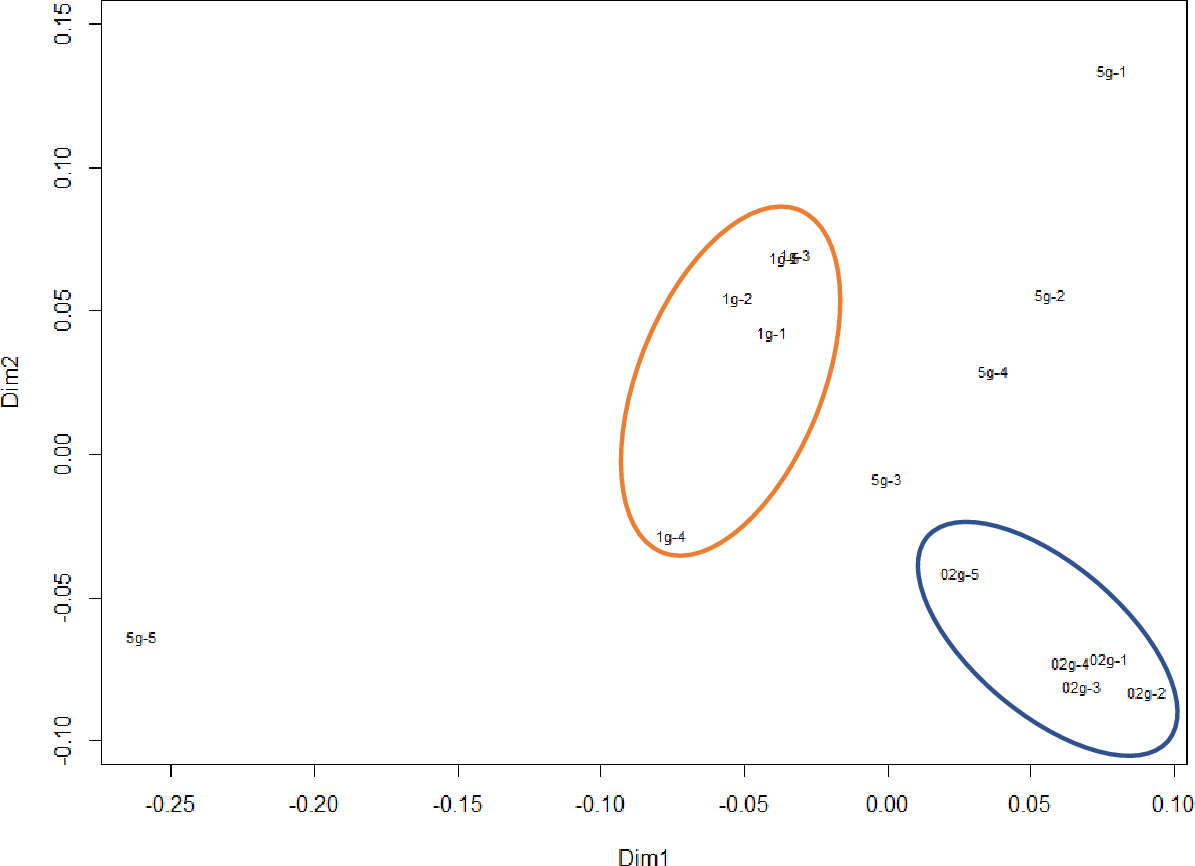

Supplement: S3 Fig — Aggregation points of the 0.2 g soil sample size are shown in blue (right) circle and those of the 1 g soil sample size are shown in orange (left) circle. Points outside the circles are for the 5 g soil sample size. 2D stress was 0.14. (TIF) [file pone.0260121.s003.tif]

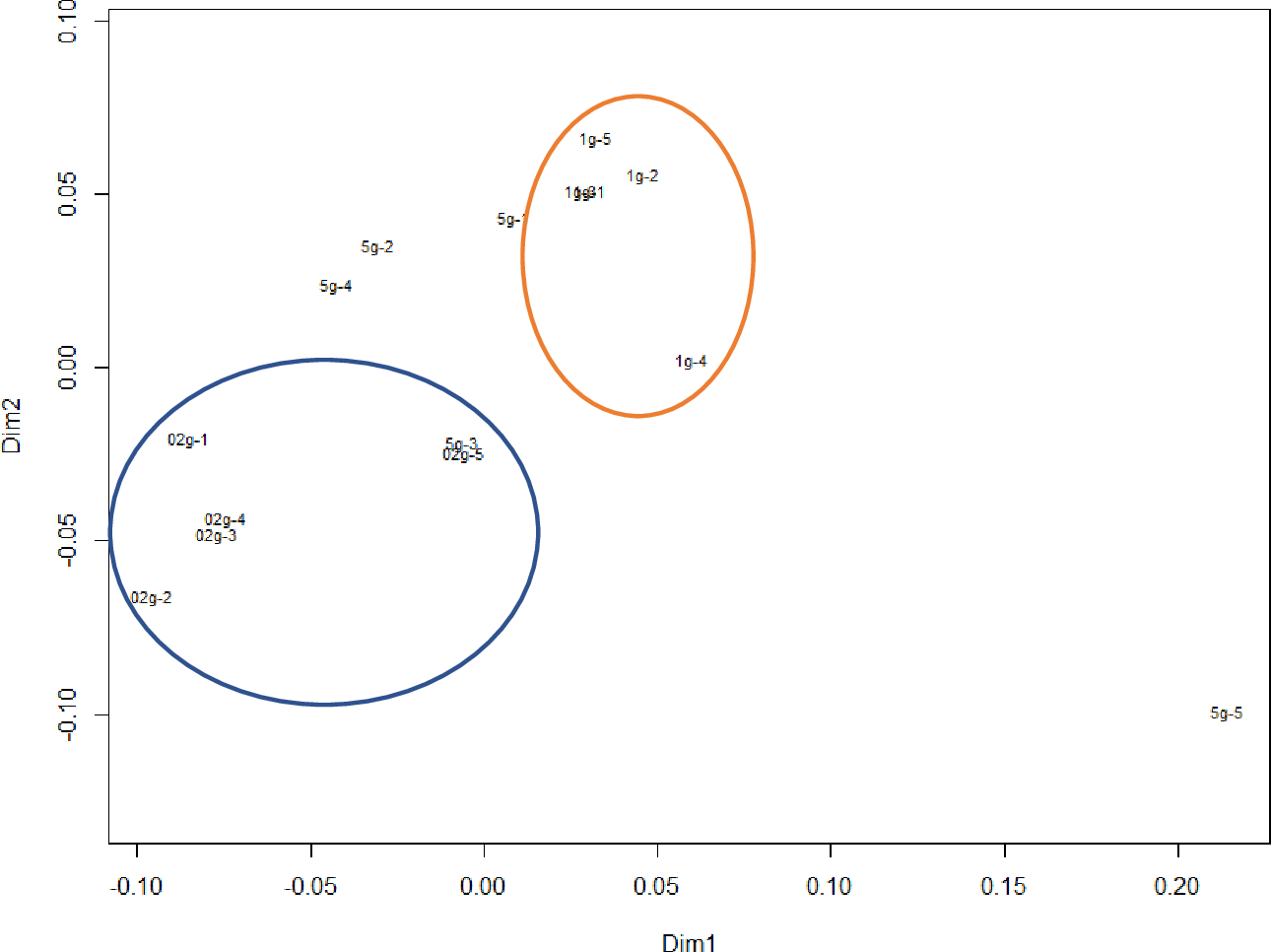

Supplement: S4 Fig — Agglomeration points of the 0.2 g soil sample size are shown in the blue (left) circle, and those of the 1 g soil sample size are shown in the orange (right) circle. Points outside the circles are for the 5 g soil sample size. 2D stress was 0.061. (TIF) [file pone.0260121.s004.tif]
