# Supplementary material for: The effect of soil sample size, for practical DNA extraction, on soil microbial diversity in different taxonomic ranks
Source: PLoS One. 2021 Nov 18;16(11):e0260121. doi: 10.1371/journal.pone.0260121 (PMC8601499; doi:10.1371/journal.pone.0260121)
Supplement: Attachment — Submitted filename: 210904_Response to Reviewers.docx [file pone.0260121.s005.docx]

Review Comments to the Author

*Reviewer #1: your work "the effect of soil sample size, for practical DNA extraction, on soil microbial diversity in different taxonomic ranks" is very interesting and useful for cauterising soil and understanding the nature of microorganisms presentation.*

Thank you very much for your encouraging words.

*Reviewer #2-1: The manuscript entitled ‘The effect of soil sample size, for practical DNA extraction, on soil microbial diversity in different taxonomic ranks' tried to determine the optimal soil sample size in regard for microbial community structure. They focused on the relationship between soil amount and DNA extraction container volume and the alteration in microbial composition at different taxonomic ranks (order, class, and phylum). Three soil sample size were tested, and they concluded that microbial diversity and diversity assessment were stable on 0.2 and 1-g samples. It is interesting to found the effect of sample size to microbial diversity. Thus, I think it is valuable to publish the paper, which would provide technical insight of sampling size.*

We appreciate the reviewer’s high evaluation.

*Reviewer #2-2: However, I would like to propose a small suggestion to the author to adjustment the conclusion. It would be easier for the reader if the conclusion is brief and concise, and which sample size practice is the best.*

Thank you very much for the useful suggestion. We have concluded that the 0.2-g soil sample level would be preferable due to its higher detection of bacteria with a high tolerance of shearing force and its economic efficiency [L. 31 and 402]. Also, we made the Abstract and the Conclusion briefer and more concise [L. 17, 27, 30, 393, and 401]. We added the following contents to explain the points mentioned (underline shows the added sentences and phrases).

- Delete: Abstract [L. 17]

To determine the optimal soil sample size for microbial community structure analysis, DNA extraction ~~(PCI reagent)~~, microbial composition analysis ~~(16S rRNA gene amplicon sequencing)~~, and diversity assessments ~~(α- and β-diversity indices)~~ were performed using soil sample sizes of 0.2, 1, and 5 g ~~(n = 5)~~.

- Delete: Abstract [L. 27]

However, non-metric multidimensional scaling showed that the microbial compositions of the two cases ~~at the order rank~~ were different~~, and such a difference was also observed at higher taxonomic ranks~~.

- Abstract [L. 30] and Conclusion [L. 401]

~~Cell grinding conditions,~~ soil sample size, and tube volume affected the estimated microbial community structures.

-> The soil sample size and tube volume affected the estimated microbial community structure.

- Abstract [L. 31]

A soil sample size of 0.2 g would be preferable to the other sample sizes because of the possible higher shearing force for DNA extraction and lower experimental costs due to smaller amounts of consumables.

- Conclusions [L. 393]

In this study, we prepared soil samples 0.2, 1, and 5 g in size, and evaluated the effect of the soil sample size on DNA extraction and microbial community structure analysis using amplicon sequence data. We focused on the relationship between the soil amount and the extraction container volume in DNA extraction, and the differences in microbial community structure between two soil sample sizes (0.2 g and 1 g) at different taxonomic ranks. In the 0.2 and 1 g soil sample sizes, the numbers of taxa and α- and β-diversity indices used were stable, with almost the same values at each taxonomic rank. However, NMDS showed that the microbial compositions of the two cases at the order rank were clearly different, and the difference was also observed at higher taxonomic ranks. The soil sample size and tube volume affected the estimated microbial community structure. A 0.2 g soil sample size would be preferable to larger sample sizes because of the possible higher shearing force for DNA extraction and lower costs for practical use with smaller amounts of consumables. When the taxonomic rank was changed from order to phylum, some minor taxa identified at the order rank were integrated into major taxa at the phylum rank. The integration affected the value of the Sørensen–Dice index. Therefore, the microbial community structure analysis was influenced by the taxonomic rank applied.

- Conclusions [L. 402]

A 0.2 g soil sample size would be preferable to larger sample sizes because of the possible higher shearing force for DNA extraction and lower costs for practical use with smaller amounts of consumables.

*Reviewer #3-1: General comments*

*This manuscript reports on the optimal soil sample size for microbial community structure analysis. Besides, this study also focused on the relationship between soil amount and DNA extraction container volume and the alteration in the microbial composition at different taxonomic ranks. The results suggest that microbial community structure analysis is influenced by the taxonomic rank applied.*

*However, I have some concerns about some of the methodology used in this study. For example, the sample sizes used and also the number of genes used in the microbial composition analysis. These indirectly impacts the general results and findings of this study.*

Thank you very much for your meaningful comments. As the reviewer mentioned, the soil sample size might affect the microbial composition analysis. Also, we have focused on the relationship between the soil sample size and the DNA extraction conditions (e.g., the volume of the container). Regarding the study using amplicon sequence data, Penton et al. and Song et al. have studied the soil sample size effect. However, However, Penton et al. evaluated the effect of the soil sample sizes (1, 5, and 10 g) using the DNA extraction kit for 10 g of soil, with the result that a larger soil sample size would be preferable. In this respect, each DNA extraction kit would have an optimal soil volume, and the smaller soil sample size in the study might deviate from the optimal volume. In other words, the relationship between the soil volume and the container volume for DNA extraction would be important, as well as the soil sample size effect, and we have investigated the points. Song et al. also elucidated the DNA extraction efficiency using the different soil sample sizes (0.25 g and 10 g) with each optimal-size DNA extraction kit, respectively. There was no significant difference in the OUT richness between the two soil sample sizes, and this result would support our idea, meaning the relationship between the soil volume and the container volume is also essential. Therefore, we added the report by Song et al. in the reference list [L. 71].

We would have liked to carry out further soil sample size cases. However, the horizontal shaking adapter for 50 mL tube volume for the vortex mixer can only equip two tubes. It is inconvenient for practical use because we usually treat many samples at one time. Therefore, we have tried a vertical, not a horizontal shaker, which can equip 6 tubes at once, to extract DNA for practical use.

We would think that the sample size indicated by Reviewer#3 means the soil sample size. However, generally, sample size means repetition of one sample. Therefore, to distinguish soil sample volume from the so-called sample size, we added the word “soil” prior to sample size, meaning soil sample volume. Also, we apologize for the confusion. Relating to the so-called sample size, we also added the reason for our repetition number of one sample (n = 5) by referring to Song et al. (2014) and Vestergaard et al. (2017) [L.50 and 117].

The number of genes also would affect microbial composition analyses. We think the reviewer’s indication has two meanings: one is the volume of sequence reads, and the other is the equality of the sequence reads by rarefaction. For the former points, 30,000-40,000 reads were obtained through the experimental protocols in our study. Therefore, the sequence reads would be enough to evaluate microbial composition analysis (Schöler et al., 2017). Also, we rewrote the sequence reads of Penton et al. from its total reads (over 5,000,000 reads) to the reads of each sample (over 56,000 reads) for the comparison of our sequence reads [L. 69]. For the latter points, we used the same soil, not the different soil. So, the number of sequence reads was one of the consequences of comparing the different soil sample sizes for DNA extraction. Therefore, we did not rarefy the read number and conducted the microbial community analysis using the obtained read number. We mentioned the point in the revised manuscript [L. 193]. By the way, the average number of sequence reads in each soil sample size would be confirmed to be almost the same (no significant difference) by applying ANOVA [L. 161]. We added the following contents to explain the mentioned points (underline shows the added sentences and phrases).

- The whole article

sample size -> soil sample size

- Introduction (L. 50)

This allows us to obtain representative and reliable results for the entire soil sampling area [6, 7], although it is time-consuming and costly.

- Introduction (L. 69)

Furthermore, minor taxa could be stably detected even in the case of the largest soil volume because of the large number of sequence reads, with an average of over 56,000 reads.

- Introduction [L. 71]

Song et al. [11] also investigated the effect of soil sample size (0.25 and 10 g) for fungal amplicon sequence data.

- Materials and Methods, Soil Sampling and DNA Extraction [L. 117]

The number of repetitions per sample (n = 5) was determined based on previous reports [7, 11].

- Materials and Methods, Statistical analysis [L. 161]

ANOVA was performed on the average number of non-chimeric sequence reads for each soil sample size (0.2, 1, and 5 g).

- Results and Discussion , Amplicon Sequencing [L. 193]

After quality filtering and chimera removal, we obtained 604,543 reads, with a range of 29,043–45,888 reads per sample. These numbers are adequate for achieving acceptable coverage [17]. The non-chimeric reads were a consequence of differences in DNA extraction conditions for the same soil sample. In addition, there was no significant difference in the average number of non-chimeric reads among the soil sample sizes by ANOVA; therefore, we used the obtained non-chimeric reads directly for the following analyses.

*Reviewer #3-2: Moreover, the authors need to clarify the novelty of their study as the same study has been shown in other research, Penton et al., 2016, even though it has been cited in the study. The authors should make this clear in the introduction and make a more comprehensive conclusion.*

Thank you for your advice. Our main discussion points are as follows; 1) effects of the target taxonomic rank and annotated taxa in microbial diversity analysis and 2) the relationship between soil volume and tube volume for DNA extraction. Firstly, we summarized the section of our motivations in an orderly way (firstly, secondly, finally) in the Introduction section [L. 76, 88, and 98]. Then, we noticed that the explanation of the former discussion point, especially using annotated taxa for microbial diversity analysis, had not been adequate in the original manuscript, so we described the point in more detail [L. 73 and 108]. We added concerns about using OTUs not annotated taxa for microbial diversity analysis [L. 76]. Also, we referred to the report by Song et al. (2015) to support our discussion [L. 71, 95]. By the way, we found our misunderstanding about the target taxonomic rank in the study by Penten et al. (2016). They did not determine a specific taxonomic rank. We apologize and corrected the point [L. 76].

We also discussed the importance of setting target taxonomic rank and using annotated taxa in microbial diversity analysis in the Results and Discussion section. We added some ideas for the transition of the Sørensen-Dice index with lowering the taxonomic rank relating to the detection of minor taxa. In addition, we wrote the possible reason for the different conclusions about detecting minor taxa between ours and Penton et al.’s. [L.339 and 348]. We also added the reference that discussed soil sample size for the detection of minor taxa [L. 355].

We re-evaluated our results for a more straightforward conclusion and concluded that a 0.2-g soil sample size would be better than a 1-g soil sample size because the soil sample size could detect taxa with higher physical shear tolerance and was carried out at a lower cost. We rewrote the choice of the two soil sample sizes in the Results and Discussion section (Determination of Sample Size) [L. 364] and the Conclusions section [L. 402]. In the Determination of Sample Size section, we summarized the advantages of each soil sample size (0.2 and 1 g) [L. 363].

We added the following contents to explain the points mentioned (underline shows the added sentences and phrases). In addition, we cleared some information in the Introduction that did not relate to the main discussing points to avoid redundancy [L. 43]. Also, we summarized the Conclusion to make it more concise [L. 393].

- Delete: Introduction [L. 43]

~~Close relationships between the surroundings and microbial communities suggest that a microbial community analysis can indicate various environmental problems. Leddicoat et al. [3] that the microbial community structure analysis could be used as a bacterial indicator of specific soil contamination and its recovery. Kepler et al. [4] investigated the effects of herbicides on crop-related microorganisms, focusing on the transition of microbial communities in the presence of the herbicide glyphosate.~~

- Introduction [L. 71]

Song et al. [11] also investigated the effect of soil sample size (0.25 and 10 g) for fungal amplicon sequence data.

- Introduction [L. 73]

Although the study by Penton et al. [10] was insightful, in our opinion, it can benefit from further clarifications, such as those concerning target taxonomic rank and annotated taxa in the microbial diversity analysis, the relationship between soil volume and tube volume for DNA extraction, and DNA extraction cost and efficiency.

- Introduction [L. 76]

First, the focused taxonomic rank and related annotated taxa might affect microbial diversity analysis. Penton et al. [10] did not describe the targeted taxonomic rank nor the annotated taxa used for microbial diversity analysis. Regarding the taxonomic rank, a taxon belonging to a minor category in a lower taxonomic rank (e.g., order) might be integrated into a major category in an upper taxonomic rank (e.g., phylum). In other words, rare taxa in lower taxonomic ranks might not be able to be classified or recognized as minor taxa in the upper taxonomic ranks. Thus, the recognition of rare taxa would depend on the target taxonomic rank. For the annotated taxa, there would usually be numerous operational taxonomic units (OTUs) not belonging to any taxa in an upper taxonomic rank (e.g., phylum) from amplicon sequence data. If these OTUs are counted as many independent pseud-taxa, the microbial diversity analysis would be biased by these OTUs. Therefore, we think that the target taxonomic rank and the annotated taxa used should be clearly defined.

- Introduction [L. 88]

Second, the relationship between soil volume, reagent volume, and tube volume may affect the efficiency and quality of DNA extraction.

- Introduction [L. 95]

Song et al. [11] also used each kit for DNA extraction from 10 g and 0.25 g of soil, respectively. There was no significant difference in OTU richness between the two soil sample sizes, possibly because of the appropriate addition of soil volume into each kit.

- Introduction [L. 98]

Finally, using a 50-mL tube for DNA extraction is expensive and inefficient; the number of tubes used for the extraction at one instance is limited if we use the recommended attachment providing horizontal vibration (Vortex Adapter for 2 (50 ml) tubes, QIAGEN) for the extraction kit.

- Introduction [L. 108]

Microbial community structure analysis at different taxonomic ranks using the annotated taxa from the amplicon sequence data was also carried out to evaluate the effect of the taxonomic rank applied on the analysis.

- Results and Discussion, Changing Taxonomic Rank [L.339]

In other words, the dispersion of taxa with lower taxonomic hierarchy would occur if a wide variety of taxa sequences exist in the samples. Given the dispersion, the value of the Sørensen–Dice index of the 1 g soil sample size at order rank should be large, at least larger than that of the 0.2 g soil sample size, because the 1 g level value was larger at the higher hierarchy (phylum) rank. However, the index value was lower than that of the 0.2 g sample size at the order rank, meaning the 1 g soil sample size might not include a wide variety of taxa sequences, especially those of minor taxa.

- Results and Discussion, Changing Taxonomic Rank [L.348]

One reason for this contradiction would be the taxonomic rank used in their analysis. Penton et al. [10] did not describe the taxonomic rank they used; however, if they used a high taxonomic rank, such a higher taxonomic hierarchy, it might not be appropriate for the detection and discussion of rare species because of the characteristic of integration.

- Results and Discussion, Changing Taxonomic Rank [L.355]

In their review, Gołębiewski and Tretyn [23] summarized that small soil sample sizes (e.g., 0.25 g) are appropriate for studying microbial diversity.

- Results and Discussion, Determination of Sample Size [L. 363]

In the previous sections, two soil sample sizes, 0.2 and 1 g, were weighed for the metagenomic analysis of soil microbial community structure. Although both sample sizes had advantages and disadvantages, we concluded that a 0.2 g soil sample size is recommended, especially for practical use. Regarding the 0.2 g soil sample size, a wide variety of taxa, including those with resistance to shear stress, were detected (Fig 2), possibly because a vortex mixer gave a higher rated power output per unit soil weight, and the smaller soil sample size was more sensitive to the hot spot effect. The values of the diversity indices, except for the Sørensen–Dice index, were almost the same between the 0.2 and 1 g soil sample sizes at different taxonomic ranks. In addition, the 0.2 g soil sample size requires smaller amounts of reagents and consumable goods for DNA extraction, making the operation economical. In contrast, the 1 g soil sample size presented stable microbial community structures at the lower taxonomic rank (order rank, Fig 10). The DNA extraction efficiency was better than that of the 0.2 g soil sample size (Fig 1). With the continued improvements in the performance of taxonomic databases and analytical techniques, an analysis of microbial community structure at a lower taxonomic rank, such as species rank, would be possible. In such a case, under the possible dispersion of taxa with the lowering of the taxonomic hierarchy, presenting a stable structure would also be required, and a larger soil sample size would be appropriate in that case.

- Results and Discussion, Determination of Sample Size [L. 364]

Although both sample sizes had advantages and disadvantages, we concluded that a 0.2 g soil sample size is recommended, especially for practical use.

- Conclusions [L. 393]

In this study, we prepared soil samples 0.2, 1, and 5 g in size, and evaluated the effect of the soil sample size on DNA extraction and microbial community structure analysis using amplicon sequence data. We focused on the relationship between the soil amount and the extraction container volume in DNA extraction, and the differences in microbial community structure between two soil sample sizes (0.2 g and 1 g) at different taxonomic ranks. In the 0.2 and 1 g soil sample sizes, the numbers of taxa and α- and β-diversity indices used were stable, with almost the same values at each taxonomic rank. However, NMDS showed that the microbial compositions of the two cases at the order rank were clearly different, and the difference was also observed at higher taxonomic ranks. The soil sample size and tube volume affected the estimated microbial community structure. A 0.2 g soil sample size would be preferable to larger sample sizes because of the possible higher shearing force for DNA extraction and lower costs for practical use with smaller amounts of consumables. When the taxonomic rank was changed from order to phylum, some minor taxa identified at the order rank were integrated into major taxa at the phylum rank. The integration affected the value of the Sørensen–Dice index. Therefore, the microbial community structure analysis was influenced by the taxonomic rank applied.

- Conclusions [L. 402]

A 0.2 g soil sample size would be preferable to larger sample sizes because of the possible higher shearing force for DNA extraction and lower costs for practical use with smaller amounts of consumables.

*Reviewer #3-3: Hence, I have to reject this manuscript because it does not meet the PLoS ONE criteria*

*However, these studies still have an impact of results in its own way*

** Consider carefully proofreading – there are several grammatical errors and also replicate of a sentence.*

Thank you for your advice. We have again discussed our novelty and the differences from previous reports and revised the manuscript as we mentioned above. We have sincerely read the revised manuscript and used the English proofreading service again.

*Other changes to the manuscript and reference list*

We added some information about the specs (e.g., manufacture, model number) of the devices and reagents [L. 90, 100, 119, 121, 127, 139, 140, and 142].

- Introduction

[L. 90] the PowerMax Soil DNA Isolation Kit (QIAGEN, Venlo, Netherlands)

[L. 100] attachment providing horizontal vibration (Vortex Adapter for 2 (50 ml) tubes, QIAGEN)

- Materials and Methods

[L. 119] the phenol/chloroform/isoamyl alcohol (PCI, 25:24:1) reagent ((Nippon Gene, Tokyo, Japan )

[L. 121] glass beads with a diameter of 1.5–2.5 mm (BZ-02，AS ONE, Osaka, Japan )

[L. 121] skim milk (FUJIFILM Wako Chemicals, Osaka, Japan )

[L. 127] centrifugation at 10,000 × g for 2 min (CF15RXII, Hitachi Koki, Tokyo, Japan)

[L. 139] a thermal cycler (MyCycler TM 170-9703JB, BIO-RAD, Hercules, USA)

[L. 140] bovine serum albumin (Takara Bio, Kusatsu, Japan )

[L. 142] (Bioengineering Lab., Sagamihara, Japan, https://gikenbio.com/)

We removed S-Fig 1 and S-Fig 2 because the readers can search these devices using their specs. So instead of these figures, we showed their specs (Table 1) [L. 122].

- Materials and Methods, Soil Sampling and DNA Extraction [L. 122]

The tube was shaken at high speed for 5 min using a mixer with its attachment (Table 1).

We had written incorrect information about the manufacture’s name of the attachment providing horizontal vibration in the original manuscript. We apologize and corrected the manufacture’s name [Table 1].

- Table 1

QIAGEN -> Scientific Industries

We had not written the version of the EzBioCloud 16S database in the original manuscript. We apologize and added the information [L. 146].

- Materials and Methods, 16S rRNA Gene Amplification and Amplicon Sequencing [L. 146]

The EzBioCloud 16S database (version 20200225) was used to detect OTUs with 97% similarity [13].

The report by Penton et al. and other reports did not describe brief instructions of the diversity indices. Therefore, we removed the description for easy reading following the relating articles [L. 157].

- Delete: Materials and Methods, Microbial Community Structure Analysis [L. 157]

~~These indices are briefly illustrated as follows:~~

~~Shannon-Wiener index:~~

$$H^{'}=-\sum_{i=1}^{S} P_{i}・\log_{2} P_{i} \left( 0\leq H^{'} \right),$$

~~where S is the total number of taxa, and Pi is the proportion of total sample representers by taxa.~~

~~Bray-Curtis dissimilarity:~~

$$B=\frac{\sum_{i=1}^{n} \left| X_{ij}-X_{ik} \right|}{\sum_{i=1}^{n} \left| X_{ij}+X_{ik} \right|}$$

~~where X_ij is the number of OTUs in sample j, and X_ik is the number of OTUs in sample k.~~

~~Sørensen-Dice index:~~

$$SDC\left( A,B \right)=\frac{(S_{A}+S_{B}-2\times S_{AB})}{\left( S_{A}+S_{B} \right)},$$

~~where SA and SB are the number of taxa constituting sample A or B. SAB is the number of taxa commonly present in both samples A and B.~~

We added a phrase for easy understanding to explain the difference of shear stress between the case of 0.2- and 1-g soil sample sizes [L. 300].

- Results and Discussion, Visualizing Community Structure [L. 300]

Although the difference in physical shear stress could also be derived from the type of mixer and the shape of containers, using small amounts of soil for DNA extraction, i.e., larger rated power output per unit soil weight, could provide an improved consequence with intense grinding.

We revised the Acknowledgment following the PLOS ONE formatting guidelines.

- Delete: Acknowledgements

~~This study is supported by “The Sanitation Value Chain: Designing Sanitation Systems as Eco-Community Value System” Project (Project Leader: Prof. Taro Yamauchi) at Research Institute for Humanity and Nature (RIHN, Project No. 14200107).~~ We would like to thank Editage (www.editage.com) for English language editing.
